# Dose optimization of vancomycin in obese patients: A systematic review

**DOI:** 10.3389/fphar.2023.965284

**Published:** 2023-03-24

**Authors:** Mahmoud E. Elrggal, Abdul Haseeb, Manal AlGethamy, Umar Ahsan, Zikria Saleem, Areej Sultan Althaqafi, Sattam Saad Alshuail, Zohair Ahmad Alsiddiqi, Muhammad Shahid Iqbal, Albaraa Faraj Alzahrani, Abdullmoin AlQarni, Rozan Mohammad Radwan, Ameer Khalid Saab Qul, Ahmad Jamal Mahrous, Jumana Majdi Alsharif, Mayyasah Khalid Alqurashi, Hani Saleh Faidah, Mohammed Aldurdunji

**Affiliations:** ^1^ Department of Clinical Pharmacy, College of Pharmacy, Umm AL-Qura University, Makkah, Saudi Arabia; ^2^ Department of Infection Prevention and Control Program, Alnoor Specialist Hospital Makkah, Makkah, Saudi Arabia; ^3^ Department of Pharmacy Practice, Faculty of Pharmacy, Bahauddin Zakariya University, Multan, Pakistan; ^4^ Department of Internal Medicine, Alnoor Specialist Hospital Makkah, Makkah, Saudi Arabia; ^5^ Department of Clinical Pharmacy, College of Pharmacy, Prince Sattam Bin Abdulaziz University, Alkharj, Saudi Arabia; ^6^ Alnoor Specialist Hospital Makkah, Department of Infectious Diseases, Makkah, Saudi Arabia; ^7^ Pharmaceutical Care Department, Alnoor Specialist Hospital Makkah, Department of Infection Prevention and Control Program, Makkah, Saudi Arabia; ^8^ Department of Microbiology, Faculty of Medicine, Umm AL-Qura University, Makkah, Saudi Arabia

**Keywords:** dose optimization, vancomycin, obese population, pediatrics, adults

## Abstract

**Background:** Dose optimization of vancomycin plays a substantial role in drug pharmacokinetics because of the increased incidence of obesity worldwide. This systematic review was aimed to highlight the current dosing strategy of vancomycin among obese patients.

**Methods:** This systematic review was in concordance with Preferred Items for Systematic Reviews and Meta-Analysis (PRISMA) guidelines. The literature search was carried out on various databases such as Scopus, PubMed/MEDLINE, ScienceDirect and EMBASE using Keywords and MeSH terms related to vancomycin dosing among obese patients. Google Scholar was also searched for additional articles. The English language articles published after January, 2000 were included in this study. The quality of the study was assessed using different assessment tools for cohort, and case reports.

**Results:** A total of 1,029 records were identified. After screening, 18 studies were included for the final review. Of total, twelve studies are retrospective and remaining six are case-control studies. A total of eight studies were conducted in pediatrics while remaining studies were conducted in adult population. Most of the studies reported the dosing interval every 6–8 h. Differences in target trough concentration exist with respect to target ranges. The administration of loading dose (20–25 mg/kg) followed by maintenance dose (15–25 mg/kg) of vancomycin is recommended in adult patients to target therapeutic outcomes. Moreover, a dose of 40–60 mg/kg/day appears appropriate for pediatric patients.

**Conclusion:** The initial dosing of vancomycin based on TBW could be better predictor of vancomycin trough concentration. However, the clinical significance is uncertain. Therefore, more studies are needed to evaluate the dosing strategy of vancomycin in overweight or obese patients.

## Introduction

Obesity (defined as a body mass index [BMI] of 30 kg/m^2^ or higher) was once considered as a minor problem while adjusting doses of medication due to limited population of obese patients (Smit et al., 2020). But, now-a-days, obesity plays a substantial role in drug pharmacokinetics (PK) because of the increased prevalence of obesity around the globe. It has been well documented that due to pathophysiological changes related to obesity, such as impacted metabolic enzyme activity, increase in adipose tissues, renal dysfunction, increased cardiac output, and pharmacokinetic parameters of the drug, there is a significant impact on healthcare system, thus requiring a dosing optimization (Knibbe et al., 2015; Smit et al., 2018). In 2008, World Health Organization (WHO) reported that approximately 1.5 billion people aged above 20 years were obese of whom 200 million were males and about 300 million were females (Grace, 2012). According to WHO, approximately 60% of the global population i.e. 3.3 billion people will be classified as obese (1.1 billion people) or overweight (2.2 billion people) by the year of 2030 (Frühbeck et al., 2013).

When calculating medication dosages for obese patients, healthcare professionals need to take into account the patient’s body weight and body composition (Alessa et al., 2021; Haseeb et al., 2021; Haseeb et al., 2022a; Haseeb et al., 2022b). There are several types of weights that can be used in dose calculations for obese patients (Cies et al., 2012; Pai, 2021). Actual Body Weight (ABW) is the patient’s current weight in kilograms (kg). ABW is often used as the initial weight when calculating medication doses for obese patients. Ideal Body Weight is the weight that a person of the same height and gender would be if they had a “normal” body mass index (BMI) of 25 kg/m2. IBW is often used as a reference weight when calculating medication doses for obese patients. Adjusted Body Weight (AdjBW) is a weight that takes into account the patient’s actual weight and their ideal body weight. AdjBW is calculated using IBW and ABW. Total Body Weight (TBW) is a weight that takes into account the patient’s total body mass, including fat, muscle, and other tissues. TBW is often used when calculating medication doses for drugs that are distributed throughout the body. The choice of weight used in medication dose calculations for obese patients depends on the medication being administered, the patient’s overall health status, and other individual factors. It is important for healthcare professionals to use appropriate weights and dosages to ensure safe and effective medication therapy for obese patients (Bearden and Rodvold, 2000; Cies et al., 2012; Schwartz et al., 2016; Pai, 2021).

For decades, vancomycin, a first glycopeptide antibiotics was used to treat various infections caused by gram-positive bacteria, especially methicillin-resistant *staphylococcus aureus* (MRSA) (Mühlberg et al., 2020; Alghamdi et al., 2021). It is considered as a first-line therapy for the treatment of MRSA infections (Choo and Chambers, 2016). Despite of having numerous benefits of vancomycin, clinicians face different challenges regarding its dosing that achieves a maximum therapeutic concentration and area under curve (AUC) to minimum inhibitory concentration (MIC) ratio that is optimal for bactericidal activity, while reducing the side effects (Cusumano et al., 2020). The standard dose of vancomycin based on total body weight is 15–20 mg/kg that is to be administered every 8–12 h in a patient with normal renal function (Liu et al., 2011). Dosing and monitoring guidelines emphasize on achieving steady-state trough concentrations. Troughs should be at least 10 μg/mL, with targets of 15–20 μg/mL for microorganism with MIC ≥1 μg/mL and for patients with serious infectious diseases (Rybak et al., 2009). These guidelines recommend dosing strategy and monitoring of vancomycin treatment in obese patients in a manner similar to non-obese patients (Durand et al., 2018). Multisite studies have been documented the PK parameters of vancomycin in non-obese patients (Katip et al., 2016; Ma et al., 2020; Arjangpour et al., 2022). However, the data among obese patient is limited. Dose optimization of vancomycin in obese patients is becoming of greater importance in the light of public health data reporting increased prevalence of obesity around the globe. This systematic review highlights the current dosing strategy of vancomycin and its clinical outcomes in obese patients.

## Materials and methods

### Data sources and searches

A comprehensive systematic review was in concordance with Preferred Reporting Items for Systematic Reviews and Meta-Analysis (PRISMA) guidelines on December, 2021 to identify relevant articles (Moher, 2015). We performed an online search in databases such as Scopus, PubMed/MEDLINE, Science Direct, and EMBASE and the Cochrane Library Central Register of Controlled Trial databases with a time limit from January, 2000 to December, 2021. Two of the reviewers performed manual research in the reference lists of included studies. Moreover, the grey literature (e.g., Google Scholar) was also searched. The search terms included *“pharmacokinetic parameters”, “dose optimization”, “vancomycin”, “dosing schedule”, “case-control”, “pediatrics”, “adults”, “overweight”, “body mass index” “obese patients”, “obesity”.* Moreover, the corresponding Medical Subject Headings (MeSH) terms were also searched. The complete search strategy for grey literature research and databases is available in Supplementary Tables S1, S2.

### Inclusion and exclusion criteria

The studies retrieved from grey literature and databases were merged and the duplicates were identified and removed using EndNote X9. The screening process was carried out in two steps. First, two of the reviewers independently screened the titles and abstracts and selected the relevant articles. Second, the selected titles and abstracts were then reviewed and validated by a third reviewer. The full-text articles were retrieved and screened by all authors for the final inclusion. The studies were included if they have met the inclusion and exclusion criteria. Studies were included if 1) they had reported about the dosing of vancomycin 2) assessed the therapeutic monitoring of vancomycin 3) published after January, 2000 4). The restriction of English language was imposed, while abstracts from conference proceedings were excluded from this study. The selected articles included was cohort studies, and case-control studies. This review also include studies in which normal-weight patients were compared with obese or overweight patients. Two reviewers (AS and ZS) independently screened the retrieved articles. In case of any disagreement, the final decision was made by third reviewer.

### Data extraction

The standardized data collection form was utilized to extract data on the studies’ metadata (e.g., author names and year, design), patients’ characteristics (e.g., sample size, clinical condition of patient), drug administration (e.g., dosing schedule of drug), clinical outcomes and recommendation for future use. Three reviewers (SD, ZS and MG) retrieved aforementioned data from the articles. The disagreements were resolved by fourth reviewer.

### Quality assessment

To critically appraise the selected articles, Newcastle-Ottawa scale (NOS) checklist were implemented for case-control and cohort studies (Wells, 2014). This scale categorizes the methodological quality of papers into three sub-scale i.e., selection, comparability and outcomes. The possible scores of NOS range from 0 to 8 for case-control and cohort studies. Any discrepancy while assessing the quality of studies was resolved by (ZS and WQ).

## Results

### Study characteristics

**FIGURE 1:**
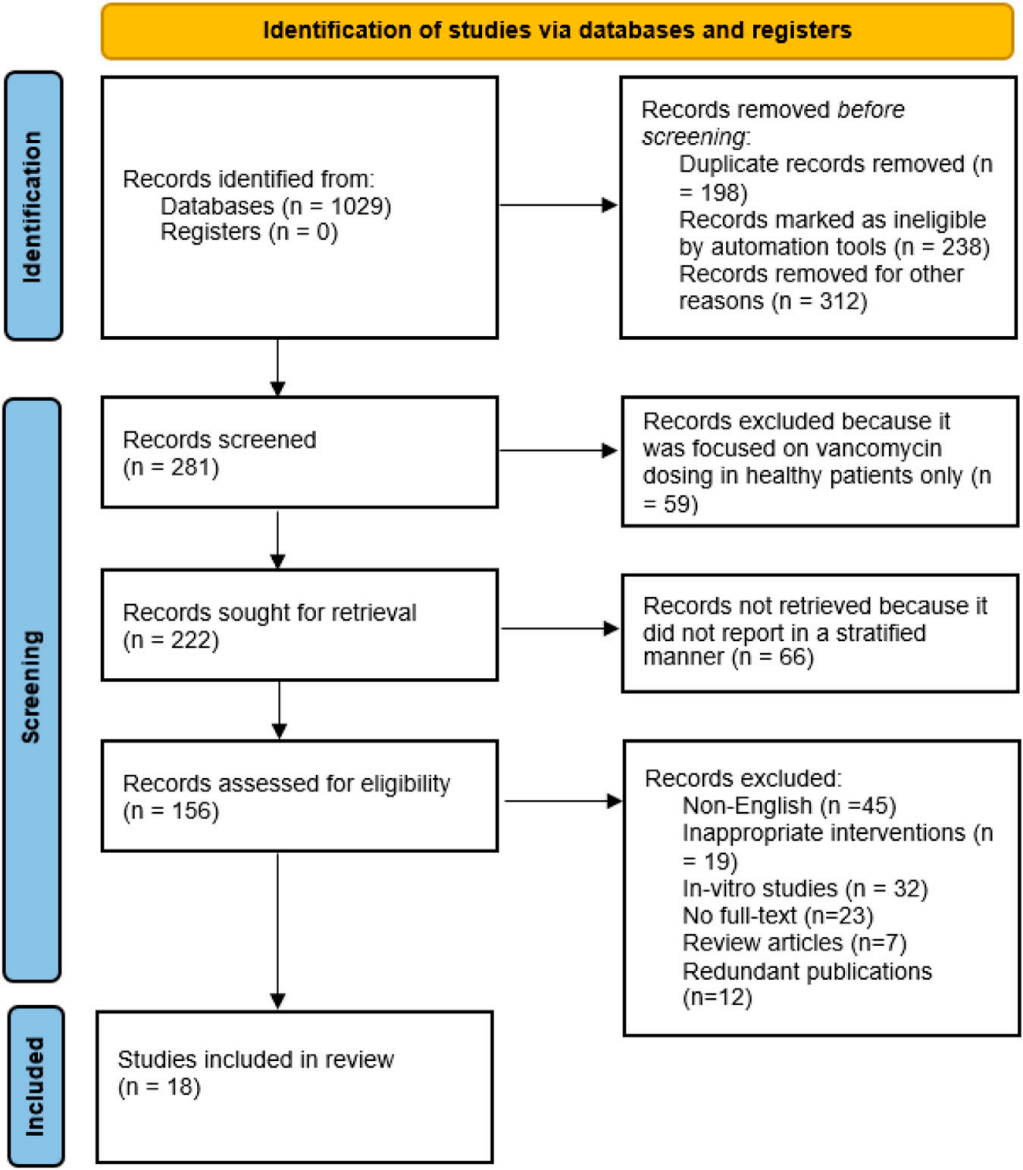
Flow chart of included studies.

A total of 1,029 articles were searched using Scopus, PubMed/MEDLINE, Science Direct, and EMBASE and the Cochrane Library Central Register of Controlled Trial databases. Following the removal of duplicate studies, the abstracts of 281 studies were screened on the basis of exclusion and inclusion criteria. After the screening process, most of the articles were excluded due to following reasons: non-English (N = 45), *in-vitro* studies (N = 32), no full-text available (N = 23), inappropriate interventions (N = 19), literature reviews (N = 7) and redundant publications (N = 12). Based on inclusion criteria, eighteen studies published in English language were included in this systematic review (Hall et al., 2008; Miller et al., 2011; Moffett et al., 2011; Nassar et al., 2012; Reynolds et al., 2012; Heble et al., 2013; Madigan et al., 2013; Eiland and Sonawane, 2014; Adane et al., 2015; Hong et al., 2015; Kosmisky et al., 2015; Kubiak, 2015; Le et al., 2015; Morrill et al., 2015; Richardson et al., 2015; Lin et al., 2016; Smit et al., 2020; Smit et al., 2021). All required characteristics of studies were presented in Table 1 and Table 2. Of total, twelve studies are retrospective and remaining six are case-control studies. A total of eight studies were conducted in pediatrics while remaining studies were conducted in adult population. The PRISMA flow diagram reporting the procedure of selection of studies is shown in Figure 1.

**TABLE 1:** Dosing strategy of vancomycin in pediatrics.

Author and Year	Design	Sample size	Characteristics of patients	Dosing strategy	Clinical outcomes	Findings
Smit et al. (2021)	Retrospective cohort study	1892	Patients with multiple infections	Patients received 15–20 mg/kg BD, TID or QID/day as a 60-min infusion.	2 compartment model significantly increase with IBW and Cl_cr_, V_c_ and V_d_	The recommended dose of vancomycin is 15 mg/kg QID daily by specifying dose reduction for renal impairment.
Le et al. (2015)	Case-control	87 normal	—	Patients received 40 mg/kg/d to 100 mg/kg/d every 6 h, 8 and 12 h.	Statistical difference in Cl of vancomycin was observed between normal weight and overweight or obese groups	Further studies are needed to evaluate the dosing strategy of vancomycin.
		87 obese				
Eiland and Sonawane (2014)	Case-control	48 healthy pediatrics	Patients with complicated infections	Patients with normal weight received mean of 10.4 mg/kg/d while patient with obesity and overweight received 14.1 mg/kg/d	There was no significant difference statistically between normal weight and overweight or obese groups.	Alternate dosing strategy is required to achieve target attainment and improve clinical outcomes
		50 obese or overweight pediatrics				
Heble et al. (2013)	Retrospective case control	42 obese patients compared with 84 normal patients.	Patient with skin, soft tissue, pulmonary, febrile, neutropenia sepsis	Dose based on Age:	Initial trough concentration of vancomycin above 20 μg/mL occurred more often in overweight and obese patients.	Special attention to TDM is narrated in all children.
				2–8 years→ 20 mg/kg/dose every 6 h		
				9–13 years→ 20 mg/kg/dose every 8 h		
				14–18 years → 15 mg/kg/dose for 2 h		
				Dose based on weight:		
				19–25 kg→ 80 mg/kg/day		
				25–50 kg→ 60 mg/kg/day		
				50–70 kg→ 45 mg/kg/day		
Madigan et al. (2013)	Case-control	222 patients	Patients with multiple infections	Patients received 40 mg/kg/d (10 mg/kg/d every 6 h) and after changes in protocol the received 60 mg/kg/d (15 mg/kg/d every 6h)	No statistical significance in trough level was observed between normal weight and obese groups.	Dose should be adjusted according to renal function and indication along with age and weight.
Nassar et al. (2012)	Retrospective cohort study	26 overweight patients	Patients with catheter associated blood stream infections, fever with neutropenia and pneumonia	20 mg/kg twice daily	No statistical difference was shown between normal, overweight and underweight patients	PK parameters were same in all groups. Moreover, higher doses are required to achieve the goals of current guidelines.
		34 normal patients				
		15 underweight				
Miller et al. (2011)	Retrospective case control	56 obese patients	Patients with multiple infections	The mean initial dose of vancomycin in 658.4 ± 389.6 mg	Reduction in odds of achieving therapeutic concentration was observed in obese patients	Regimen after every 8 h was considered as appropriate dosing schedule.
Moffett et al. (2011)	Retrospective case control	24 obese patients	Patients with multiple infections	Patients received 14.1 + 1.5 mg/kg every 6 h, 8 h or 12 h	Higher trends were observed in vancomycin trough concentration	Overweight/obese patients should receive vancomycin based on ABW.

IBW, Ideal body weight; QID, Four times daily; BD, Twice daily; TID, Thrice daily; PK, Pharmacokinetic; CLcr, Creatinine clearance; Vd, Volume of distribution; TDM, Therapeutic drug monitoring; PK, Pharmacokinetic; ABW, Actual body weight.

**TABLE 2:** Dosing strategy of vancomycin in adults.

Author and Year	Design	Sample size	Characteristics of patients	Dosing strategy	Clinical outcomes	Findings
Reynolds et al., 2021 (Reynolds et al., 2012)	Retrospective	138 obese patients	Patients with multiple infections	The mean ± SD of MD was 19 ± 2 mg/kg/day with revised protocol and 34 ± 7 mg/kg/d with original protocol	The revised protocol resulted in a higher frequency of target troughs.	Revised protocol of vancomycin dosing improved the attainment of target trough concentration.
Smit et al. (2020)	Prospective	20 obese patients	Patients with bariatric surgery	12.5 mg/kg, maximum 2500 mg/day received infusion during and immediately surgery.	A dose of 35 mg/kg/day, maximum 5500 mg/day resulted in >90% target attainment	Vancomycin should be dosed as 35 mg/kg/day (maximized at 5500 mg/kg/day).
Lin et al. (2016)	Retrospective	18 obese patients	Patients with multiple infections	Weight based daily maintenance dose 25.6 mg/kg in obese and 43.8 mg/kg in non-obese patients	Mean daily maintenance dose required to achieve a level of 20 mcg/mL were 2,961 ± 1,670 mg in obese compared to 3,189 ± 1,600.69 mcg in non-obese	Critically ill obese patients treated with vancomycin required low maintenance dose/unit BW to achieve same target level
		26 non-obese patients				
Richardson et al. (2015)	Retrospective cohort study	37 obese patients	Patient with multiple infections	A dose of 15 mg/kg IBW with a maximum initial dose of 2000 mg	Increasing obesity predicted higher probability of SVCs >20 mg/L	Development of alternative dosing and management strategies for vancomycin is required.
		71 non-obese patients				
Kosmisky et al. (2015)	Retrospective	48 obese patients	Patients with multiple infections	LD based on TBW	35.4% patients showed the attainment of therapeutic trough concentration	Further studies are needed to assess the optimal dosing strategy of vancomycin in obese patients.
				Critically ill: 25 mg/kg		
				Non-critically ill: 20 mg/kg		
				MD based on Renal function		
				CL_cr_ (>65) 10 mg/kg Q12 h		
				CL_cr_ (35–65) 10 mg/kg 24 h		
				CL_cr_ (<35 or dialysis) 10 mg/kg renal dosing		
Morrill et al. (2015)	Retrospective	263 obese patients	Patients with suspected MRSA and pneumonia	The mean total DD 2005 ± 736 mg in obese and 2,306 ± 934 mg in extremely obese administered.	20% patients achieved optimal target trough concentration	30 mg/kg/day was considered as appropriate dose for obese patients and 20–25 mg/kg/day for extremely obese patients.
		71 extremely obese patients				
Kubiak (2015)	Retrospective	67 obese patients	Patients with suspected MRSA	Patients received dose of vancomycin of 45–65 mg/kg/day	73% patients receiving dose based of IBW had initial vancomycin trough concentration of <15 mg/L	Further PK studies are required for the optimal dosing strategy of vancomycin.
Adane et al. (2015)	Prospective	31 obese patients	Patients with suspected and confirmed MRSA	Patients received dose of vancomycin of 4,000 mg/day	Stimulations reported that 4,000—5,000 mg/day of vancomycin had more than 93% probability of target attainment	Vancomycin dosing strategy can be altered on the basis of renal function.
Hong et al. (2015)	Retrospective and Prospective	150 obese patients	Patients with pneumonia, sepsis, endocarditis and meningitis	MD dose of vancomycin will be given at maximum dose of 2000 mg.	32% in pre-intervention and 42.7% in post-intervention groups achieved initial therapeutic trough concentration achieved while in patients with second trough measurement, 31.0% in pre-intervention and 65.2% in post-intervention group were within the therapeutic range.	Measurement of 2 serum vancomycin concentration significantly improves subsequent target trough concentration attainment in the obese population.
				LD will be administered at maximum dose of 3,000 mg as per hospital policy.		
Hall et al. (2008)	Prospective	155 obese patients	Patients with Gram positive bacterial infections	Patients received 15 mg/kg/dose for obese	Adequate initial dosing was achieved for 27.7% of obese.	Obese patients received inadequate vancomycin dosing. Greater efforts should be taken to ensure patient receive weight-based dosing.

IBW, Ideal body weight; SVC, Serum vancomycin concentration; MD, Maintenance dose; LD, Loading dose; PK, Pharmacokinetic; CLcr, Creatinine clearance; DD, Daily dose; TBW, Total body weight.

### Dosing strategy of vancomycin in pediatrics

In children, the recommended dose of vancomycin is 15–20 mg/kg/dose every 6 h. Majority of the studies reported the dosing interval every 6–8 h. However, in a Nassar et al., study, patients received dose of vancomycin twice daily (Nassar et al., 2012). A study reported that a dose of 20 mg/kg every 8 h was considered as an appropriate dosing schedule for obese pediatrics with multiple infections (Miller et al., 2011). However, another study suggested a dose of 15/mg/kg/dose twice daily by specifying dose reduction for renal dysfunction in patients with various multiple infections (Smit et al., 2021). Nassar and his colleagues reported that no statistical difference was shown among normal, overweight and obese patients with catheter-associated infections who received dose of 20 mg/kg/dose twice daily (Nassar et al., 2012). Moffett et al., reported that PK parameters were not altered in patients who received a dose of 14 mg/kg every 6 h or 8 h or 12 h (Moffett et al., 2011). However, in a pharmacokinetic model performed by Le and his colleagues documented lower clearance of vancomycin in overweight and obese patients using Bayesian estimation method (Le et al., 2015). Differences in target trough concentration exist with respect to target ranges 10–15 ug/mL, 10–20 ug/mL and 15–20 ug/mL in each included study.

### Dosing strategy of vancomycin in adults

In adults, the recommended dose of vancomycin is 15–20 mg/kg/day every 8–12 h. Adane and his colleagues conducted a prospective study in which patients with suspected MRSA infections received dose of 4,000 mg/day and >90% probability target attainment was achieved using aforementioned dose (Adane et al., 2015). However, in retrospective study, authors recommended the dose of 45–65 mg/kg/dose on the basis of ideal body weight (IBW) in the patients with suspected MRSA infections (Kubiak, 2015). About 73% of patients had an initial trough concentration less than 15 mg/L (Kubiak, 2015). Similarly, another study reported that the dose of 15 mg/kg/dose given on the basis of IBW resulted in higher probability of serum vancomycin concentration (SVC) greater than 20 mg/L (Richardson et al., 2015). Another prospective study reported that the appropriate dose of vancomycin is 35 mg/kg/day in patients with bariatric surgery (Smit et al., 2020). Reynolds and his colleagues retrospectively compared the original protocol (a loading dose of 20–25 mg/kg, followed by the maintenance dose of 15 mg/kg every 8–12 h) with revised protocol (a loading dose of 20–25 mg/kg, followed by maintenance dose of 10 mg/kg/day (Reynolds et al., 2012). He concluded that the revised protocol improved the attainment of target trough concentration.

### Quality assessment

The quality of the study was considered higher when scored ≥7, moderate when scored 5-6, and low when scored <5. A total of 6 studies were awarded 8 stars while the remaining studies were awarded 7 stars. The stars awarded for studies ranged from 7-8 and the average value was 7.7 (Table 3).

**TABLE 3:** Quality assessment of cohort studies

	Selection				Comparability	Outcomes		
References	Representative of exposed studies^A^	Selection of non-exposed^B^	Ascertainment of exposure^C^	Demonstration of outcome^D^	Comparability of cohort studies on basis of design^E^	Assessment of outcomes^F^	Adequacy of follow-up^G^	Quality score
Smit et al. (2021)	*	*	*	*	*	*	*	7
Reynolds et al. (2012)	*	*	*	*	*	*	*	7
Smit et al. (2020)	*	*	*	*	*	*	*	7
Lin et al. (2016)	*	*	*	*	**	*	*	8
Hong et al. (2015)	*	*	*	*	*	*	*	7
Richardson et al. (2015)	*	*	*	*	**	*	*	8
Kosmisky et al. (2015)	*	*	*	*	*	*	*	7
Morrill et al. (2015)	*	*	*	*	*	*	*	7
Kubiak (2015)	*	*	*	*	*	*	*	7
Adane et al. (2015)	*	*	*	*	*	*	*	7
Le et al. (2015)	*	*	*	*	**	*	*	8
Eiland and Sonawane (2014)	*	*	*	*	**	*	*	8
Heble et al. (2013)	*	*	*	*	**	*	*	8
Madigan et al. (2013)	*	*	*	*	*	*	*	7
Nassar et al. (2012)	*	*	*	*	**	*	*	8
Miller et al. (2011)	*	*	*	*	*	*	*	7
Moffett et al. (2011)	*	*	*	*	*	*	*	7
Hall et al. (2008)	*	*	*	*	*	*	*	7

## Discussion

However, literature exists for altered PK parameters of vancomycin in obese patients, the clinical practice with vancomycin dosing gives challenges to healthcare professionals. In this systematic review, we found that differences exist in the dosing strategy of vancomycin in pediatrics as well adult population with overweight and obesity. The evidence on PK studies in obese adults describes the correlation between the volume of distribution and total body clearance with total body weight (TBW) (Bearden and Rodvold, 2000; Leong et al., 2011; Alobaid et al., 2016). Instead of using adjusted or ideal body weight, the recommended dosing strategy for obese adult population is to utilize TBW to achieve therapeutic outcomes (Khare et al., 2020). The administration of loading dose (20–25 mg/kg) followed by maintenance dose (15–25 mg/kg) of vancomycin is recommended in adult patients to target therapeutic outcomes (Al-Kathiri et al., 2021). However, it remains unclear if changes in metabolism such as absorption, volume of distribution and clearance are different in pediatrics with overweight compared with pediatric with obesity because the majority of the articles studied these patients in one group.

According to IDSA guidelines, the recommended dose of vancomycin is 15 mg/kg/dose every 6 h in pediatrics and 15–20 mg/kg/dose every 8–12 h for the treatment of MRSA infections (Liu et al., 2011). The IDSA also suggested the AUC/MIC ratio of vancomycin should be ≥400, which corresponds to the target trough serum concentration of 15–20 μg/mL in adult population (Giuliano et al., 2010). A target trough concentration of 15–20 μg/mL was also recommended for pediatrics with serious infections such as endocarditis, bacteremia, pneumonia, meningitis, osteomyelitis and skin infections (Liu et al., 2011). These suggestions are based on limited data obtained from case-control studies, reports of expert committees and clinicians’ experience (Durand et al., 2018). We found that most of studies conducted in pediatrics did not follow the IDSA-recommended dosing strategy of 15 mg/kg/day every 6 h (Nassar et al., 2012; Madigan et al., 2013; Le et al., 2015). Therefore, further studies are required to examine the pediatric population with obesity and overweight taking higher doses of vancomycin. Several studies have been previously reported that the differences exist in trough concentration associated with target exposure due to age, weight or dosing frequency in obese as well as normal-weight patients. Therefore, healthcare practitioners should not adjust the dose based on trough concentration alone but preferably utilize the Bayesian estimation method to correlate the therapeutic drug monitoring (TDM) samples to identify exposure, as is also suggested in the modified TDM guidelines of vancomycin (Frymoyer et al., 2014; Janssen et al., 2016; Rybak et al., 2020).

Alternative dosing strategies of vancomycin have been introduced recently. Some studies suggested that a maintenance dose of 60 mg/kg/day according to TBW and the utilization of Bayesian estimation method of dosing showed the highest achievement of an AUC/MIC ratio of ≥400 in obese pediatrics with suspected or confirmed MRSA infections (Le et al., 2013; Le et al., 2014). A continuous dosing regimen of vancomycin resulted in an improved target attainment concentration when compared with the intermittent infusion of vancomycin in infants with sepsis (Gwee et al., 2019). Similarly, the adult population receiving the continuous infusion of vancomycin showed significantly lower incidence of nephrotoxicity than patient treated with intermittent infusion (Hao et al., 2016). Wesner and his colleagues introduced a nomogram on the basis of weight and creatinine clearance to calculate the dose of vancomycin for achieving serum vancomycin concentration (SVCs) level in obese population (Wesner, 2013). However, limited data were found on nephrotoxicity associated with higher vancomycin dosing. Higher tough serum concentration results in an increase in the risk of nephrotoxicity in overweight and obese children, although this concern needs further evaluation (Choi et al., 2017; Fiorito et al., 2018).

Further PK studies are required to understand the differences in current dosing protocols and to find out the clinical outcomes to evaluate the correlation between PK parameters and obesity in the pediatric and adult populations (Kyler et al., 2019). Additional and more extensive PK studies in which researchers examine AUC, V_d_, and clearance of vancomycin that should be conducted in obese patients to optimize therapeutic dosing and minimize side effects. Following the different studies regarding an increase in MRSA resistance to vancomycin, increased serum trough levels are suggested for the treatment of various severe or invasive infections (Kullar et al., 2011; Leu et al., 2012; Elyasi et al., 2014; Almangour et al., 2017; Almangour et al., 2019). Therefore, future vancomycin PK studies should also be focused strictly on the obese population with severe infections such as endocarditis, bacteremia, MRSA infection, pneumonia, meningitis, osteomyelitis and skin infections. This would help clinicians in assessing the appropriate dosing strategy to achieve therapeutic outcomes.

This systematic review has set some limitations. Firstly, the data was extracted from small retrospective and prospective observational studies. Secondly, the sample size was generally small that may affect the outcomes. Thirdly, the majority of the studies reported dosing strategy from single-center that may reflect variability in vancomycin dosing protocol. Moreover, the clinical outcomes were not assessed as the main focus was on target trough concentration in majority of the studies, which lack prospective evidence to use this dosing protocols as a surrogate marker for clinical outcomes. The included studies did not assess the nephrotoxicity and other adverse events associated with higher doses of vancomycin in obese population.

## Conclusion

The physiological changes in patients with obesity and overweight resulted in alteration of dosing protocol of vancomycin. Our study supports the practice of administering loading dose (20–25 mg/kg) followed by maintenance dose (15–25 mg/kg) of vancomycin in obese adults while in pediatrics, a dose of 40–60 mg/kg/day appears appropriate to achieve therapeutic outcomes. The initial dosing of vancomycin based on TBW could be better predictor of vancomycin trough concentration rather than adjusted or ABW. However, the clinical significance is uncertain. Therefore, there is an urgent need for better-defined dosing strategy in obese and overweight patients. Moreover, additional studies are needed to evaluate the alternate dosing strategy in overweight or obese patients and assess how obesity and overweight affects the PK parameter to increase the efficacy, reduced side effects and promote antibiotic cost savings.
