# Supplementary material for: Dose optimization of vancomycin in obese patients: A systematic review
Source: Front Pharmacol. 2023 Mar 24;14:965284. doi: 10.3389/fphar.2023.965284 (PMC10081578; doi:10.3389/fphar.2023.965284)
Supplement: Supplementary file 1 [file DataSheet1.docx]

# Table S1. Retrieval strategy and search results from Grey Literature

| **#** | **Search terms** | **Results** |
| --- | --- | --- |
| 1 | (VANCOMYCIN [MeSH Terms]) OR (vancomycin [Text Word]) | 123,574 |
| 2 | Dose optimization [MeSH Terms] OR Dosing regimen*[Text Word] | 232,624 |
| 3 | (Obesity [MeSH Terms]) OR ((Overweight*[Text Word] | 98,652 |
| 4 | (Adults [Text Word] AND Pediatrics [Text Word] | 78,543 |
| 5 | #1 AND #2 AND #3 AND #4 | 156,162 |
| 6 | #1 AND #4 | 98,744 |
| 7 | #1 AND #2 AND #3 | 66,564 |

# Table S2. Retrieval strategy and search results from Databases

| **#** | **Search terms** | **Results** |
| --- | --- | --- |
| 1 | (VANCOMYCIN [MeSH Terms]) OR (vancomycin [Text Word]) | 185,343 |
| 2 | Dose optimization [MeSH Terms] OR Dosing regimen*[Text Word] | 65,343 |
| 3 | (Obesity [MeSH Terms]) OR ((Overweight*[Text Word] | 67,894 |
| 4 | (Adults [Text Word] AND Pediatrics [Text Word] | 45,654 |
| 5 | #1 AND #2 AND #3 AND #4 | 77,538 |
| 6 | #1 AND #4 | 87,645 |
| 7 | #1 AND #2 AND #3 | 59,231 |
